# Expression of a recombinant bacterial l-asparaginase in human cells

**DOI:** 10.1186/s13104-019-4836-5

**Published:** 2019-12-05

**Authors:** Raquel Caminha Dantas, Ludmilla Freire Caetano, Ariany Lima Sousa Torres, Matheus Soares Alves, Emanuelly Thays Muniz Figueiredo Silva, Louhanna Pinheiro Rodrigues Teixeira, Daniel Câmara Teixeira, Renato de Azevedo Moreira, Marcela Helena Gambim Fonseca, Saul Gaudêncio Neto, Leonardo Tondello Martins, Gilvan Pessoa Furtado, Kaio Cesar Simiano Tavares

**Affiliations:** 1 Experimental Biology Center (NUBEX), University of Fortaleza (UNIFOR), Fortaleza, Brazil; 2 Oswaldo Cruz Foundation (FIOCRUZ), Fortaleza, Brazil

**Keywords:** l-Asparaginase, Acute lymphoblastic leukemia, Glycosylation, Hypersensitivity

## Abstract

**Objective:**

l-Asparaginase (ASNase) is an enzyme used in the treatment of acute lymphoblastic leukemia (ALL). As the therapeutic ASNases has bacterial origin, severe side effects are associated with its use, among them hypersensitivity and inactivation of the enzyme. In this context, the objective of this work was to produce a recombinant ASNase of bacterial origin in human cells in order to determine the presence and consequences of potential post-translational modifications on the enzyme.

**Results:**

Recombinant ASNase was expressed in human cells with a molecular weight of 60 kDa, larger than in *Escherichia coli*, which is 35 kDa. N-glycosylation analysis demonstrated that the increased molecular weight resulted from the addition of glycans to the protein by mammalian cells. The glycosylated ASNase presented in vitro activity at physiological pH and temperature. Given that glycosylation can act to reduce antigenicity by masking protein epitopes, our data may contribute to the development of an alternative ASNase in the treatment of ALL in patients who demonstrate side effects to currently marketed enzymes.

## Introduction

l-Asparaginase is an important biopharmaceutical used in acute lymphoblastic leukemia (ALL) chemotherapy. This disease consists of a heterogeneous group of lymphoid neoplasms that results from monoclonal proliferation and accumulation of immature lymphoblasts in the bone marrow, peripheral blood and other organs [1].

Three formulations of ASNases are currently available on the market. Two are derived from *Escherichia coli* (native ASNase and pegylated ASNase) and one is derived from the bacterium *Erwinia chrysanthemi* [2]. Treatment with ASNase may induce undesirable effects in patients, such as blood clotting and gastrointestinal disorders, nerve symptoms, pancreatitis and liver function problems [3]. Anti-ASNase antibody formation may also be related to the development of hypersensitivity or result in silent inactivation of the enzyme and consequent treatment failure [4].

Research and characterization of ASNases in different organisms has been the focus of several studies [5–7]. An as yet little explored possibility is the glycosylation of *E. coli* ASNase by mammalian cells. Glycosylation may directly act to reduce immunogenicity of proteins [8]. In this work we demonstrate that *E. coli* ASNase is glycosylated when expressed in mammalian cells, keeping its maximal activity at similar pH and temperature to those of the non-glycosylated enzyme.

## Main text

### Methods

#### Construction of expression vectors

**Fig. 1:**
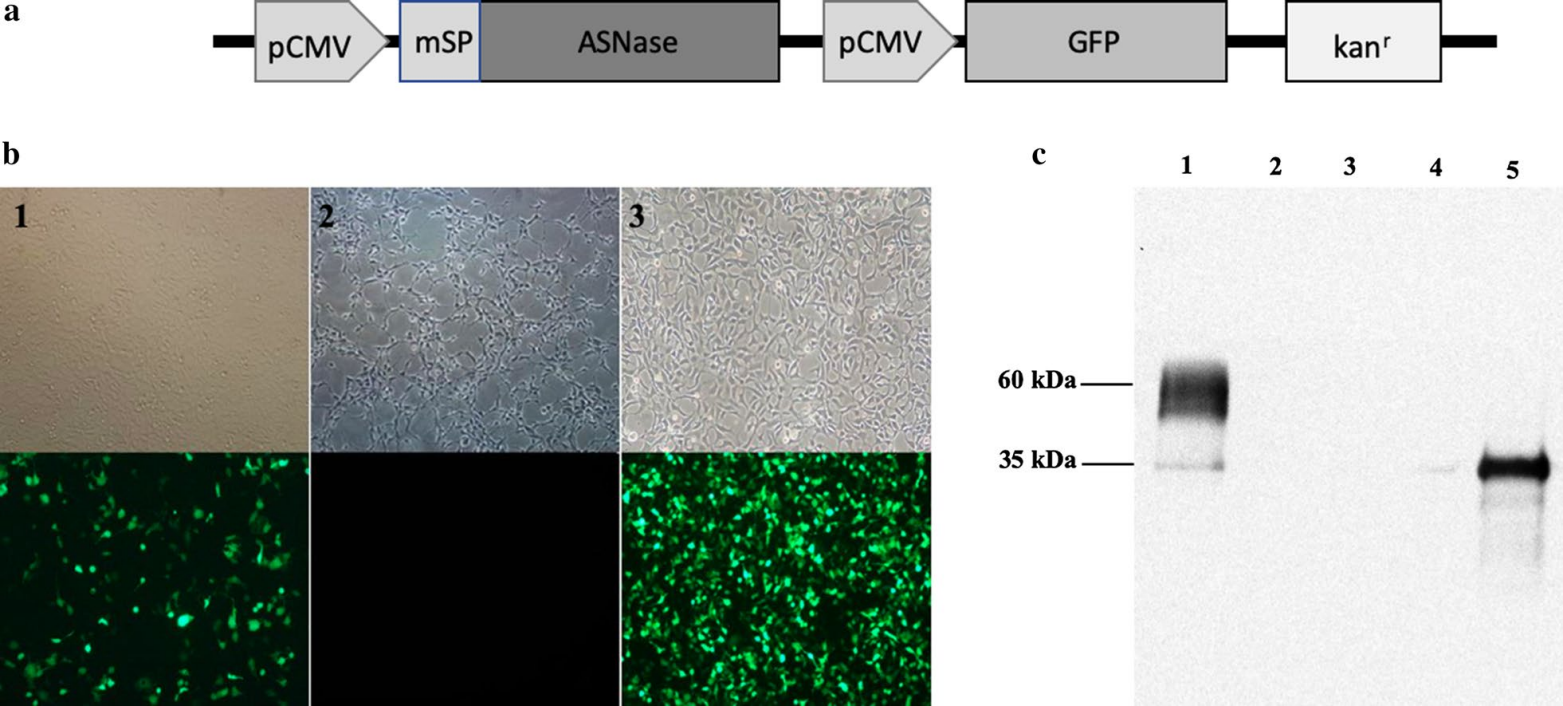
Bacterial l-asparaginase gene expression in mammalian HEK-293 cells. **a** pASNase vector, containing two in tandem CMV promoters (pCMV), the coding sequence for the *E. coli* l-Asparaginase gene (ASNase) with a milk signal peptide sequence (mSP) and a kanamycin resistance gene (kan^r^). **b** Transfection of HEK-293 cells in white light (top line) and under UV light (bottom line) with the pASNase vector in order to produce clones with the stably integrated transgene. (1) Cells transfected with the linear pASNase plasmid, (2) Cells transfected without DNA (negative control), (3) Cells transfected with a commercial GFP plasmid (positive control). **c** Immunodetection of recombinant l-Asparaginase secreted in the medium of HEK-293. (1) Cell culture medium from HEK-293 cells transfected with pASNase plasmid, (2) Cell culture medium from HEK-293 cells transfected with no DNA (negative control), (3) Cellular lysate from HEK-293 cells transfected with pASNase plasmid, (4) Cellular lysate from HEK-293 cells transfected with DNA (negative control), (5) Commercial l-Asparaginase from *E. coli* (positive control)

In order to achieve a high expression level of ASNase in HEK-293 cells, the coding sequence of the *Escherichia coli* ansB gene (GenBank Gene ID: 947454), which codes for bacterial l-Asparaginase II, had its codons optimized for expression in mammals through a commercial service (Genscript, Piscataway, USA) and was added from a coding sequence for a signal peptide that directs protein secretion in milk at its N-terminal portion (GenBank Accession Number MN435795). The sequence was then subcloned into the pAdTrack-CMV vector (Addgene # 16405). The final construct, called pASNase (Fig. 1a), was linearized with the restriction enzyme *EcoR*I for further transfection into mammalian cells.

For ASNase expression in *Escherichia coli*, the genomic DNA of *Escherichia coli* BL21 strain was used as a template to amplify the ansB coding sequence by PCR and ligated into the pET 28a (+) plasmid. *E. coli* Rosetta DE3 strain, transformed with the pET28a-ansB construct, was used for ASNase expression. The enzyme was purified through a nickel affinity column (Ni–NTA—Promega, Madison, USA).

#### Production of a mammalian cell line expressing ASNase

HEK-293 cells were electroporated with 5 µg of linear pASNase vector in 2 pulses of 1150 V and 20 mS using the Neon Transfection System (Thermo Fisher Scientific, Waltham, USA). Two days after transfection, cells were collected and redistributed into four 96-well plates at a density of 2 cells/well. Green colonies were split until a pure cell line was obtained. The insertion of the pASNase construct was confirmed by PCR and DNA sequencing.

#### Expression and detection of recombinant ASNase in mammalian cells

A HEK-293 cell clone containing ASNase was expanded to ten 100 mm plates. The medium with ASNase was collected and the recombinant enzyme was concentrated using the Vivaspin 10 MWCO (General Electric Healthcare, Boston, USA) system. Detection of ASNase expression was performed by western blot, using an anti-ASNase polyclonal antibody produced in rabbits (orb344093, Biorbyt, Cambridge, UK) and as the secondary antibody an anti-rabbit-HRP (NA 934, General Electric Healthcare, Boston, USA). The membrane was revealed through chemiluminescence using the Fluorchem FC2 (Protein Simple, San Jose, USA) equipment. As positive control, commercial *E. coli* ASNase (A3809, Sigma Aldrich) was used.

#### ASNase Glycosylation Analysis

The ASNase sequence used for expression in HEK-293 cells was analyzed for potential N-glycosylation sites with NetNGlyc 1.0 software [9]. Samples of ASNase expressed in HEK-293 and *E. coli* were digested with PNGase-F enzyme (New England Biolabs, Ipswich, USA) according to the manufacturer’s instructions. Following digestion, western blot immunodetection of the enzyme was performed as previously described in the section “Expression and detection of recombinant ASNase in mammalian cells”, using an anti-ASNase primary polyclonal antibody produced in rabbits (orb344093, Biorbyt, Cambridge, UK) and as the secondary antibody an anti-rabbit-HRP (NA 934, General Electric Healthcare, Boston, USA).

#### ASNase relative activity

The ASNase activity was assayed using Nessler´s reagent with adaptations for microscale reaction. The assay contained 10 µL of dilute enzyme, 10 µL of 189 mM l-asparagine solution and 160 µL of appropriated buffer. The reaction mixture was incubated at different pHs and temperatures for 30 min, and then was stopped by adding 10 µL of 1.5 M TCA solution. The ammonia released in the supernatant was detected using 10 µL of the reaction mixture, 25 µL of Nessler’s reagent and 215 µL of distilled water. The solution was read at 436 nm using a microplate reader with a 96-well plate. The maximum pH for the asparaginase activity was determined at 37 °C in 50 mM of various buffers in pH ranges of 3–11 (citrate–phosphate—pH 3.0–6.0; Tris pH 7.0–9.0; glycine pH 10–11). The maximum temperature was determined by incubating the reaction mixture at temperatures ranging from 20 °C to 90 °C at pH 8.0, using a thermocycler. All assays were performed in triplicate.

### Results

#### ASNase expression in mammalian cells

The efficiency of pASNase transfection in HEK-293 cells was estimated in 50%, based on 24 h GFP expression after electroporation (Fig. 1b). After limiting dilutions of transfected cells, isolation of 11 colonies expressing GFP was possible. Of these, one demonstrated intense and homogeneous GFP expression and was chosen for subsequent experiments.

Expression analysis showed that the ASNase produced by HEK-293 was secreted into the cell culture medium. Two ASNase bands were detected in the cell supernatant, one more intense at 60 kDa and one weaker at 35 kDa (Fig. 1c). Commercial *E. coli* ASNase showed an intense band at the size of 35 kDa.

#### N-Glycosylation of bacterial ASNase expressed in mammalian cells

**Fig. 2:**
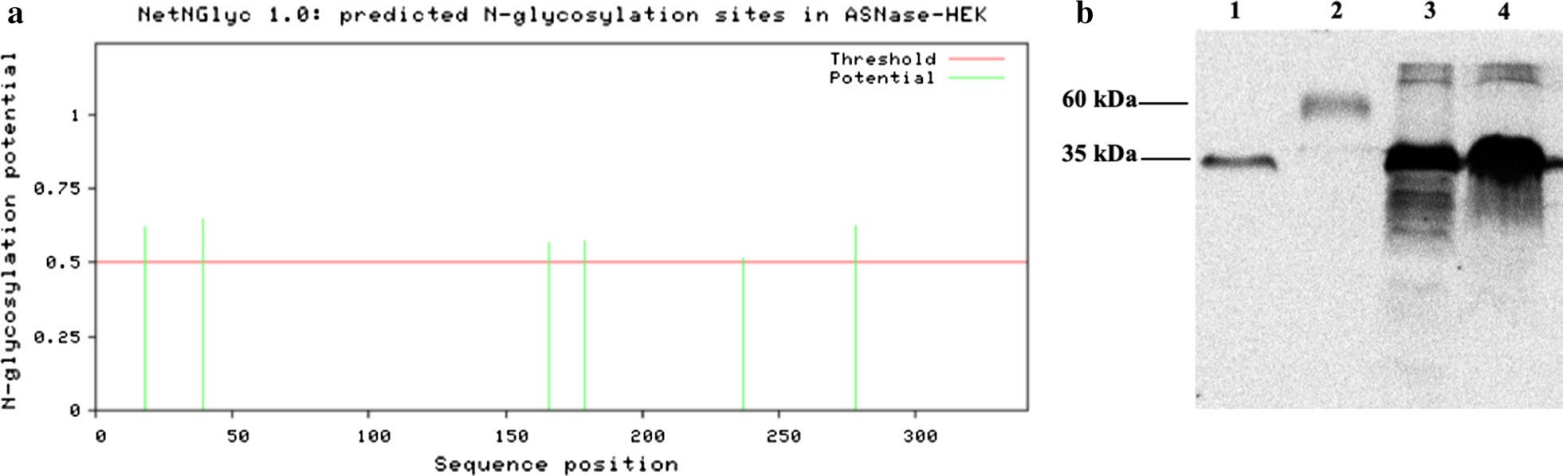
Glycosylation analysis of the bacterial l-Asparaginase expressed in the mammalian cell line HEK-293. **a** Predicted N-glycosylation sites in the l-Asparaginase sequence expressed in HEK-293 cells using the NetNGlyc 1.0 software. A position with the potential (vertical lines) crossing the threshold (horizontal line at 0.5) is predicted when glycosylated. **b** Immunodetection of recombinant l-Asparaginase expressed in HEK-293 cells and *E. coli* after deglycosylation with PNGase-F. (1) HEK-293 l-Asparaginase treated with PNGase-F, (2) HEK-293 l-Asparaginase untreated with PNGase-F, (3) *E. coli* l-Asparaginase treated with PNGase-F, (4) *E. coli* l-Asparaginase untreated with PNGase-F

The presence of a larger than expected band size for ASNase expressed in HEK-293 (Fig. 1c) evidenced the possibility of glycosylation in the enzyme. Computational analysis predicted the possibility of 6 potential N-glycosylation sites in the ASNase sequence used (Fig. 2a). To confirm the presence of glycans in the ASNase expressed in mammalian cells, the enzyme was digested with the enzyme PNGase-F, which cleaves N-glycosylations anchored to the protein backbone. The result confirmed the presence of N-glycosylation in the enzyme, which was 35 kDa in size, similar to the *E. coli* ASNase, after treatment with glycosidase (Fig. 2b).

#### Glycosylated ASNase relative activity at different pHs and temperatures

**Fig. 3:**
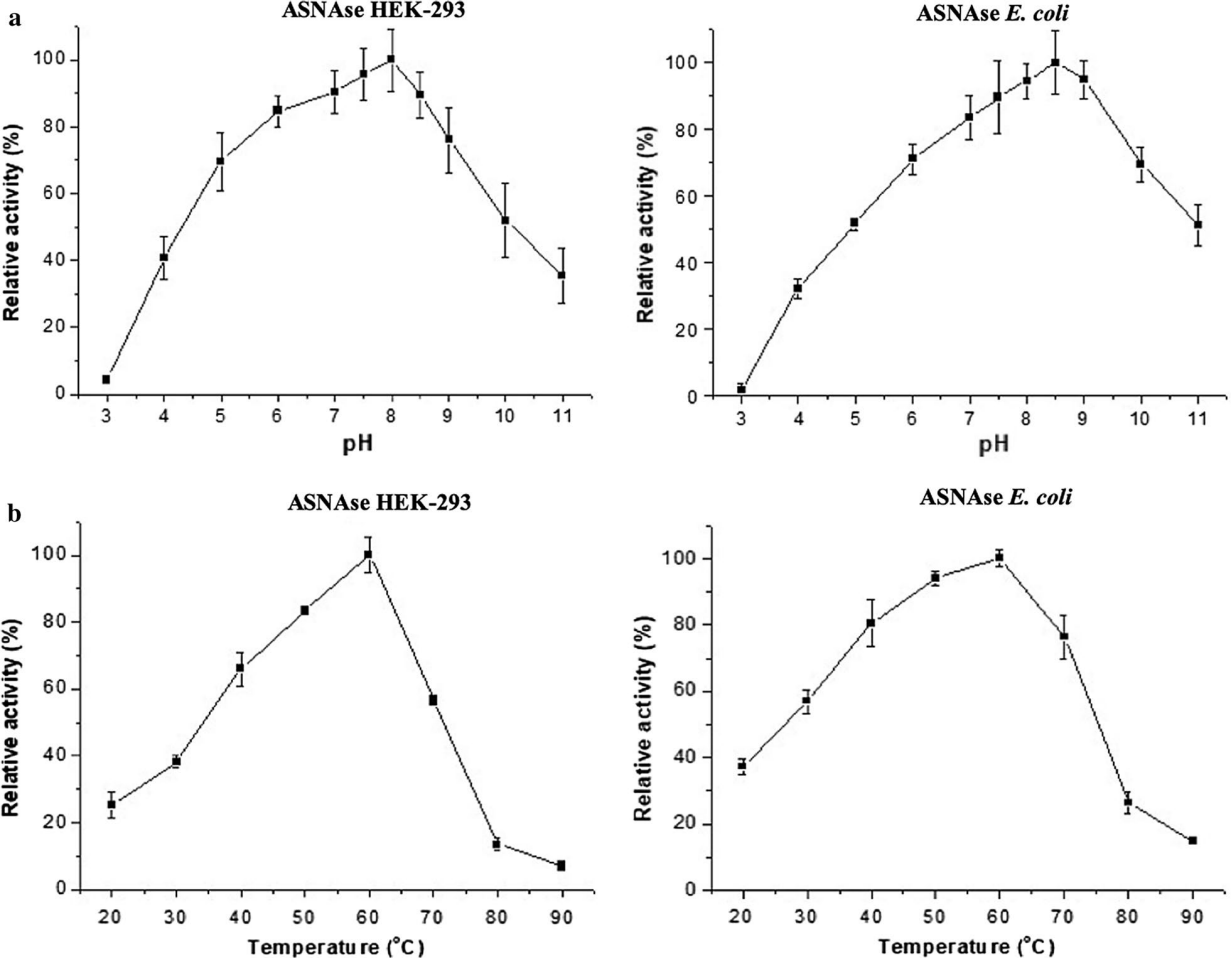
Bacterial and HEK-293 l-Asparaginase relative activity at different pHs and temperatures. **a** Effect of the pH on the activity of the glycosylated bacterial l-Asparaginase expressed in the mammalian cell line HEK-293 (left) and in *E. coli* (right). **b** Effect of temperature, at pH 8.0, of the glycosylated bacterial l-Asparaginase expressed in the mammalian cell line HEK-293 (left) and in *E. coli* (right)

l-Asparaginases expressed in *E. coli* and HEK-293 had very similar relative activity profiles at the different pH conditions and temperatures tested. Both showed a maximum activity temperature of 60 °C and a slight variation in the pH of maximum activity, with 8.0 and 8.5 for the enzymes expressed in HEK-293 and *E. coli*, respectively (Fig. 3).

### Discussion

The active form of the enzyme encoded by the *ansB* gene in *E. coli* is a tetramer, having four identical subunits of 35.6 kDa each [10]. In our results, the ASNase expressed in HEK-293 mammalian cells had two bands, one of 35 kDa and a more intense at 60 kDa, present only in mammalian cell culture medium (Fig. 1c). This difference could be associated with glycosylation, since the addition of sugars to the protein chain may alter its electrophoretic migratory pattern [11].

In fact, our results confirmed the presence of N-glycosylation in ASNase expressed in mammalian cells, since after treatment with PGNase-F, the size of the enzyme detected in western blot changed from 60 kDa to 35 kDa, the same as the ASNase expressed in *E. coli* (Fig. 2b) and is as expected for the protein backbone. l-Asparaginases from eukaryotic organisms such as fungi can also be glycosylated [12, 13]. However, it is reported that glycosylation patterns different to those in humans can trigger immune responses in patients [14].

According to our results, glycosylated *E. coli* l-Asparaginase was active under different pH and temperature conditions. Both glycosylated and native ASNases showed similar optimal relative activities at a temperature of 60 °C and a pH range between 8.0 and 8.5 (Fig. 3). Compared with commercially available ASNases, the pegylated enzyme has an optimum temperature of 50 °C and pH 7.0 [15], while the ASNase produced by *E. chrysanthemi* shows maximum activity between 30–40 °C and pH 8.5 [16, 17].

Interestingly, reports in the literature show that glycans may mask epitopes present in the protein chain. The human granulocyte colony stimulating factor produced in mammalian cells was not recognized by patient antibodies, while the same biopharmaceutical produced in bacteria and yeast was reactive [18, 19]. In addition, the incorporation of glycosylation sites in bovine beta-lactoglobulin, considered the main allergen of bovine milk, reduced its immunogenicity in mice, which was attributed to the coverage of protein epitopes by sugars [20]. In this context, our results suggest that the *E. coli* ASNase glycosylated by mammalian cells, after further studies on its antitumor activity and immunogenicity, may represent a possible alternative for ALL patients refractory to the bacterial enzyme treatment.

## Limitations

The limitations of the study were:

- We did not performed experiments to demonstrate the impact of glycosylation on immunogenicity. Our hypothesis is based only in the cited literature.
- Despite our data suggests that the ASNases from *E. coli* and HEK-293 had similar optimal relative activities at different pH and temperature, the activity assays performed in this work reports relative activity for each enzyme (from *E. coli* or HEK-293), making it not possible to directly compare the absolute activity level between them.

## Data Availability

All data generated or analysed during this study are included in this published article.

## Abbreviations

ASNase: l-asparaginase
ALL: acute lymphoblastic leukemia
PCR: polymerase chain reaction
PBS: phosphate buffered saline
GFP: green fluorescent protein
LB: Luria–Bertani
mSP: milk signal peptide sequence
TCA: trichloroacetic acid
